# Comparison of Two Preoperative Radiographic Methods for Assessing Tibial Tuberosity Advancement to Achieve a Postoperative Patella Tendon Angle of 90° in Dogs

**DOI:** 10.3390/ani13142310

**Published:** 2023-07-14

**Authors:** Federica Aragosa, Giovanni Della Valle, Chiara Caterino, Barbara Lamagna, Sara Buonocore, Francesco Lamagna, Gerardo Fatone

**Affiliations:** 1 Department of Veterinary Medicine and Animal Production, University of Naples “Federico II”, 80137 Naples, Italy; 2 Freelance Consultant, 80137 Naples, Italy

**Keywords:** modified Maquet procedure, tibial tuberosity advancement, wedge selection, cranial cruciate ligament failure, dogs

## Abstract

**Simple Summary:**

In dogs, the current techniques for the preoperative planning of tibial tuberosity advancement do not appear to restore joint stability due to under-advancement after surgery. This cadaveric study compared the tibial-anatomy-based method and the common tangent method for measuring tibial tuberosity advancement. The postoperative patellar tendon angle was not significantly different between the two methods. However, the tibial-anatomy-based method yielded advancement similar to the sizes of commercially available wedges. Both techniques yielded a tibial tuberosity advancement within the suggested range. However, TAM resulted in a mean value of the postoperative patellar tendon angle corresponding to 90°. This study paves the way for developing intraoperative methods capable of achieving a patellar tendon angle that may not be influenced by preoperative variables.

**Abstract:**

Previous studies have suggested that the preoperative methods used to plan tibial tuberosity advancement in dogs may result in under-advancement. Therefore, this cadaveric study compared the effectiveness of the common tangent method and the tibial-anatomy-based method for achieving a target patellar tendon angle (PTA) of 90° after the modified Maquet procedure. Twenty stifle joints of mesomorphic dogs were randomly assigned to the two measurement methods. Radiographs taken in the mediolateral projection were used to measure tibial tuberosity advancement, and the wedge size was selected accordingly. For each surgical procedure, a custom-made three-dimensional wedge matched to an OrthoFoam wedge was used as a spacer. Postoperative radiographs were used to measure the PTA and to evaluate the position of the wedge. The measured advancement was not significantly different between the two methods. For 60% of the cases, the advancement measured using the common tangent method was <5.3 mm and the wedge size was increased to match that of commercially available wedges. Consequently, there was a significant difference between the measurements and wedges selected between the two procedures. The postoperative PTA did not differ significantly between the two methods and was 90° ± 5° in 80% of the stifles. The position of the wedge relative to the osteotomy was not significantly different between the methods. In conclusion, the advancement determined using the tibial-anatomy-based method was generally consistent with the size of commercially available wedges, and the method yielded a mean postoperative PTA of 90°.

## 1. Introduction

Cranial cruciate ligament failure is one of the principal causes of pelvic limb lameness in dogs [1]. The cranial cruciate ligament (CCL) assumes a paramount role in stabilizing the canine stifle joint. Impairment of the CCL’s functionality leads to an instability of the stifle joint characterized by cranial tibial translation relative to the distal femoral condyles during weight-bearing, subsequently resulting in the development of osteoarthritis [2,3,4,5,6]. Rupture of the CCL constitutes a prevalent cause of non-traumatic pelvic limb lameness in dogs. This occurrence is primarily attributed to progressive degenerative rupture, commonly known as cranial cruciate ligament disease, although instances of acute traumatic rupture have also been documented [6]. The precise etiopathogenesis underlying CCL rupture remains incompletely understood, with diverse genetic, environmental, and mechanical factors exerting influence over the progression of the disease [2,4,6,7]. Some studies suggest that the initial clinical manifestation preceding complete CCL rupture is inflammation or synovitis of the stifle joint [8,9]. Once observable signs of inflammation become evident, approximately 85% of dogs subsequently experience CCL rupture [10].

Prevalence values of 0.56% to 2.6% have been reported for cranial cruciate ligament disease [11] and, surprisingly, even a value of about 11% has been reported for North American hospitals between 1994 and 2003 [1]. The treatment of choice for addressing a CCL rupture entails surgical intervention aimed at mitigating the tibiofemoral shear force and restoring functional stability to the stifle joint during limb utilization. Numerous static and dynamic procedures targeting the stifle joint have been previously documented in the literature, including tibial tuberosity advancement (TTA) and tibial plateau levelling osteotomy (TPLO) [12]. These techniques were developed to restore joint stability and slow secondary joint degeneration. Newer surgical techniques have been developed in recent decades and are grouped into tibial tuberosity advancement techniques (TTATs) [13]. The purpose of TTATs is to reduce the PTA to 90° [14]. Unlike traditional TTA methods, the newer techniques preserve the distal tibial hinge by incomplete osteotomy, which allows for the curvilinear advancement of the tibial tuberosity without proximal displacement [15,16]. Therefore, the attachment of the patellar tendon is rotated cranially but cannot move proximally, resulting in patella baja. Additionally, the extent of distal patellar displacement increases with larger cage sizes. While distal translation of the patella occurs in 15% of patients undergoing similar surgery in human medicine, no prevalence data are available in veterinary medicine [17]. The clinical implications are currently unknown, but an association between patella baja and an increased incidence of congenital lateral patellar luxation has been noted in dogs [18].

Currently, the advancement required to achieve a postoperative patellar tendon angle (PTA) of 90° is assessed on preoperative radiographs [19,20,21,22,23]. To achieve the target PTA, a cage or wedge chosen according to the advancement that is determined preoperatively is inserted into the osteotomy gap [16,24]. Therefore, measuring the intended advancement is essential when performing a TTAT. Following the introduction of TTA, several preoperative planning methods have been developed, but the discrepancy between the value of the advancement assessed on the radiographs and the true advancement obtained intraoperatively has been widely reported in the veterinary literature [15,25,26,27,28,29].

Several factors affect the measurement of the required advancement, including limb positioning, the tibial plateau angle (TPA), tibial anatomic features, femorotibial subluxation, the TTA measurement method, and the PTA assessment method [15,28,30,31,32]. Modifying the stifle angle during positioning or varying the method to measure the stifle angle will alter the PTA and, accordingly, the calculated advancement. More precisely, if the PTA is measured by a radiograph performed at a knee angle <135°, a reduced advancement of the tibial tuberosity is obtained, which is not sufficient to neutralize the cranial tibial shear force. Similarly, an angle greater than 135° results in a greater advancement of the tibial tuberosity, leading to a caudal shear force [33]. On the other hand, in 2022, Giansetto et al. measured the stifle angle in the mid-stance phase in different breeds and reported that the angle was close to an extension of 145°. Therefore, they assumed that planning surgery with the stifles positioned at an extension of 135° could cause under-advancement of the tibial tuberosity [31,34].

Currently, TTA is not recommended at a TPA greater than 31° because of the risk of severe under-advancement [25]. As the TPA increases, the measurement of advancement decreases, and a greater discrepancy between the preoperative measurements and postoperative outcome occurs [25]. The percentage of under-advancement calculated on tibial models with a mean TPA of 24.4° has ranged from 21% to 28% [26]. However, it has been suggested that tibial conformation has a greater effect on the under-advancement of tibial tuberosity compared to the TPA [27].

Among the preoperative planning methods proposed, only the tibial-anatomy-based method (TAM) does not require a stifle position at an extension of 135°, and this explains why advancement prediction is required during preoperative planning [33,34].

None of the methods used to predict the required TTA have yielded the correct advancement [15,25,26,27,28,29]. Therefore, the aim of this study is to compare the effectiveness of two preoperative radiographic planning methods, the tibial-anatomy-based method and the common tangent method (CT), to achieve a final PTA of 90° after the modified Maquet procedure (MMP) in canine patients with CCL failure.

## 2. Materials and Methods

For this study, the protocols and procedures were reviewed and approved by the Ethical Animal Care and Use Committee of the University of Naples “Federico II” (PG/2023/0080711).

Twenty stifles from adult dog cadavers (*n* = 10) of mesomorphic breeds weighing >20 kg were selected for this study. All dogs were euthanized or died for reasons unrelated to this study. After death, they were immediately transferred and stored. Dogs with a history or radiological signs of trauma or skeletal disease were excluded from the study. The stifles were stored at −20 °C and then at room temperature for 6 h before starting the study. The hindlimbs were not disjointed to preserve their biomechanical function. The right and left hindlimbs of each dog were randomly divided into two groups to undergo either CT or TAM.

**Figure 1:**
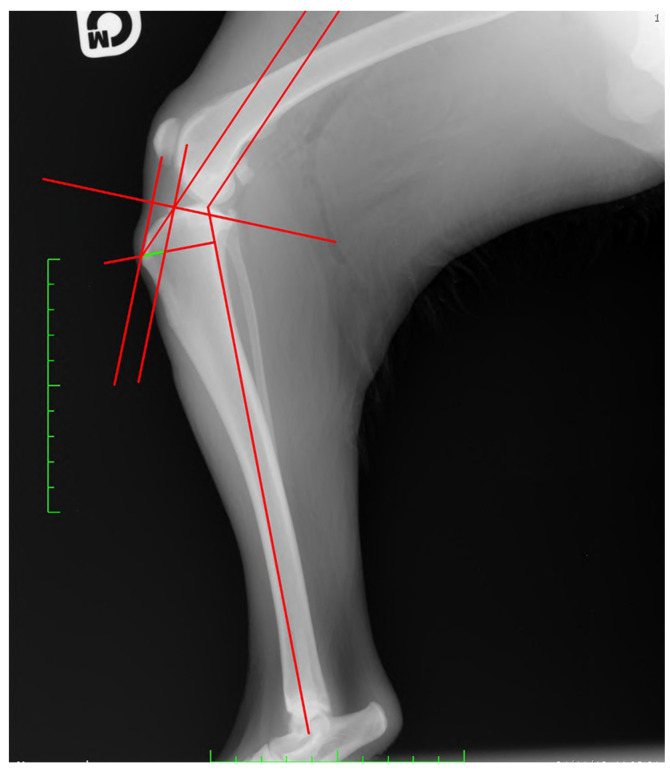
Measurement of the required tibial tuberosity advancement (green line) using the tibial-anatomy-based method.

**Figure 2:**
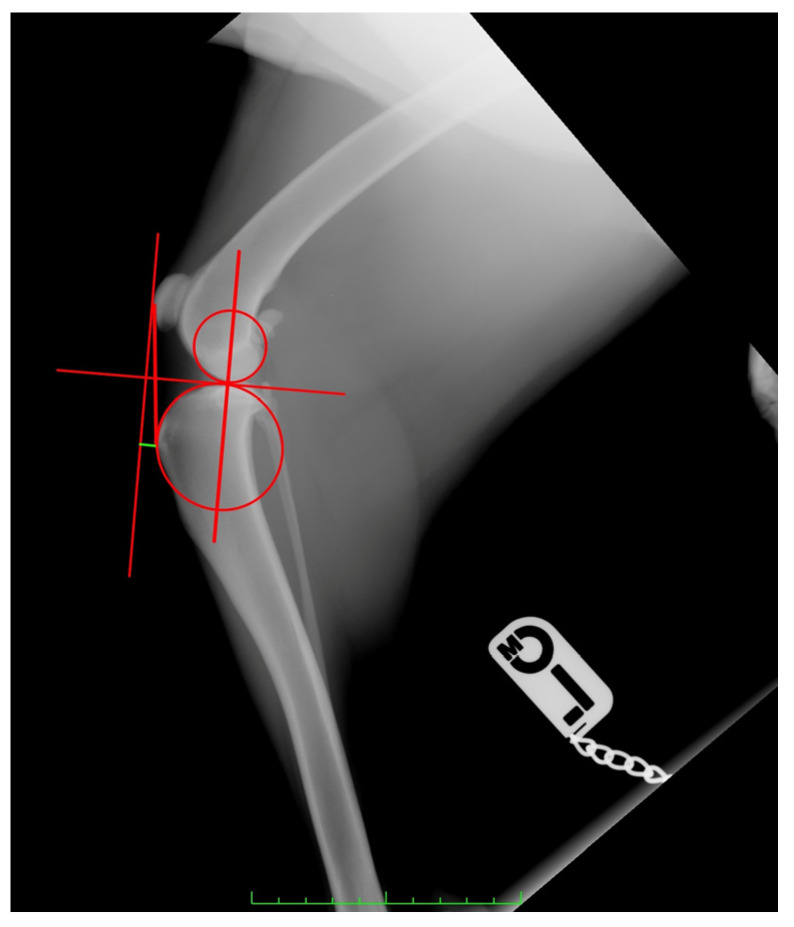
Measurement of the required tibial tuberosity advancement (green line) using the common tangent method.

For each hindlimb, a radiograph (Philosophy HF 400, I.P.S. Medical S.r.l.s., Bussolengo, Italy) was taken in the mediolateral projection. The beam was centered over the stifle with the hindlimb positioned at an angle of 135°. The position was considered correct if the image included the distal third of the femur, the intercondylar eminence, the entire tibia, and the talocrural joint and if the femoral and tibial condyles were superimposed with a gap of <2 mm on the radiographic projection. The radiographs were used to measure TTA by CT or TAM [22,35] (Figure 1 and Figure 2).

Measurement methods were applied as previously described and are summarized below:

− For CT, first, the observer drew two circles representing the femoral and tibial condyles, marking the center. Next, he connected the two centers with a line and drew a line perpendicular to it, defined as the common tangent. The angle between the common tangent and the line drawn from the caudal margin of the patella to its insertion on the tibial tuberosity corresponded to the PTA. To measure the amount of advancement required, the observer considered the distance between the tibial tuberosity and the line perpendicular to the common tangent starting from the cranial margin of the patella [35] (Figure 1).

− For TAM, the tibial functional axis of the tibia, defined by a line joining the midpoint between the intercondylar tibial tubercles (in the stifle joint) with the center of the talocrural joint, was drawn. Then, the tibial plateau, defined by a line joining the points at the cranial-most and caudal-most edges of the medial tibial condyle, was drawn. Secondly, from the functional axis, a caudally directed 135° angle towards the femur was made. Next, a parallel line through the patellar insertion point on the tibial tuberosity was located. This line intersected the tibial plateau line that was previously drawn. A perpendicular line to the tibial plateau was placed starting from patellar insertion. Next, a parallel line through the intersection point was drawn. The distance between this line and the most-cranial point of the tibial tuberosity, measured along a line perpendicular to the function axis, was recorded as the required advancement [22] (Figure 2).

All measurements were performed using an open-source DICOM viewer (Horos, version 3.3.6, 64-bit, Nimble Co LLC d/b/a Purview, Annapolis, MD, USA, https://www.horosproject.org (accessed on 4 December 2022)) by an experienced surgeon (Ph.D with 15 years of experience in orthopedic surgery), and a second observer (a third-year Ph.D student in veterinary surgery) selected the wedge size accordingly. The evaluator was unaware of the age, weight, or breed of the dogs for the radiographs he was assessing. The wedge used in the procedure was selected as the commercially available size (6 mm, 7.5 mm, 9 mm, 10.5 mm, 12 mm, and 13.5 mm) that was closest to the size determined on the preoperative radiograph.

The MMP was performed by an experienced surgeon using the method described by Ness in 2016 [22]. The limb was clipped, and a medial approach to the stifle joint was performed in order to expose the medial surface of the cranial tibia and of the femorotibial joint. A dedicated saw guide (Orthomed Ltd., Majestic House, 29 Green Street, Huddersfield, West Yorkshire, HD1 5DQ, UK) was used to perform the osteotomy of the tibial tuberosity. Progressive TTA was achieved using a distractor up to the size of the selected wedge. A custom-made polylactic acid wedge made with a three-dimensional printer (Anycubic i3 Mega, Anycubic Technology CO., Limited, Room 803, Chevalier House, 45–54 Chatham Road South, Tsim SHA TSUI, Kowloon, Hong Kong) of the same shape and size as the OrthoFoam MMP wedge was used to maintain and stabilize the TTA. Postoperative radiographs were used to measure the PTA and the appropriate position of the wedge. The preoperative and postoperative PTAs were measured using the tibial plateau method, as previously described, and reported as the mean ± standard deviation (SD) [14]. The tibial plateau slope was identified, and the PTA was measured as the angle between it and the patellar tendon axis.

**Figure 3:**
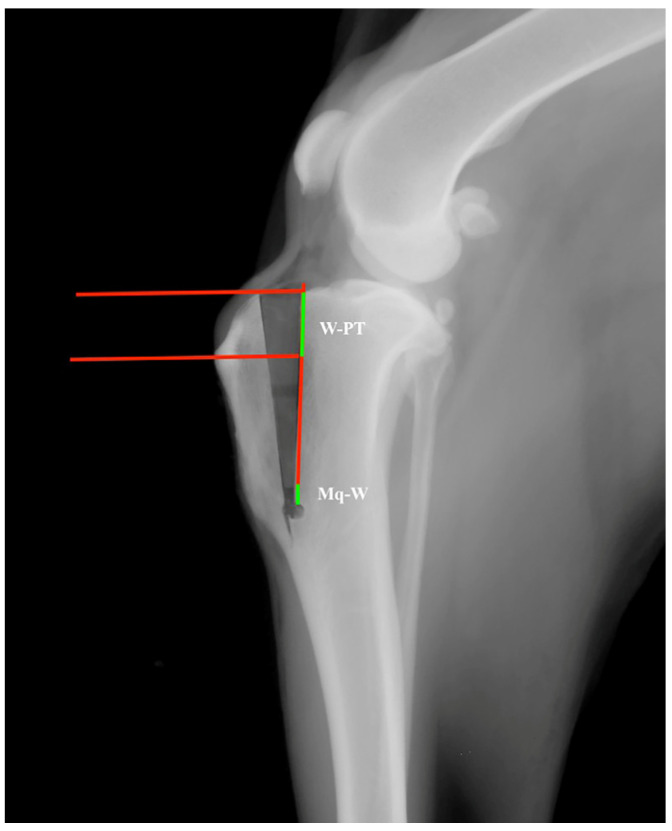
Line between the proximal edge of the wedge and the patellar tendon insertion corresponding to W-PT (green line) and line between the distal edge of the wedge and the Maquet hole corresponding to Mq-W (green line).

The correct position of the wedge (W) was assessed postoperatively by drawing a line corresponding to the osteotomy from the articular line to the Maquet hole (Mq). Then, three perpendicular lines corresponding to the proximal and distal edges of the wedge that passed through the insertion of PT at the tibial tuberosity were traced. Finally, the distance between the Maquet hole and the distal edge of the wedge (Mq-W) was measured across the osteotomy line. The segment of the osteotomy line between the proximal edge of the wedge and the insertion of the PT corresponded to W-PT (Figure 3). Both Mq-W and W-PT are expressed in millimetres.

The preoperative and postoperative PTA and TTA measurements, wedge size, Mq-W, and W-PT were recorded in a spreadsheet (Microsoft Excel 2019. Microsoft Corporation, Redmond, WA, USA) and imported into SPSS Statistics (Version 28.0. IBM Corporation, Armonk, NY) for data analyses. Normal distribution was determined using the Shapiro-Wilk test. All continuous variables are expressed as the mean ± SD and non-parametric variables as the median (range). The differences in preoperative and postoperative PTA were compared between the two methods using the Mann-Whitney test. The differences between the preoperative and postoperative PTA of TAM and CT were examined using the Wilcoxon test. The statistical significance level was set at *p* < 0.05.

## 3. Results

All dogs used in the study were mesomorphic breeds. Their mean ± SD weight was 28.8 ± 5.4 kg.

Twenty stifles were used and randomly assigned using online randomization (https://www.randomizer.org (accessed on 31 October 2022)) to two groups of ten stifles, each based on the two techniques. The mean preoperative PTA was 95° ± 4.4 using TAM and 97.6° ± 3.7° using CT. The preoperative PTA was not significantly different between the two methods (*p* = 0.173).

The mean TTA determined preoperatively was 9.3 ± 1.2 mm using TAM compared with 6.5 ± 3.2 mm using CT. There was no significant difference in the advancement calculated by the two methods (*p* = 0.07).

The mean size of the wedges determined using TAM and CT was 9.3 ± 1.2 and 8.4 ± 2.1 mm, respectively. For both methods, six wedges of 7.5 mm, six of 9 mm, four of 10.5 mm, two of 12 mm, and two of 6 mm were used. The size of the selected wedges was not significantly different between the two methods (*p* = 0.16).

Comparing the advancement values and the sizes of the selected wedges between the two methods showed a significant difference for CT (*p* = 0.013), and 90% of the chosen wedges were larger than the calculated advancement. By comparison, for TAM, the size of 30% of the selected wedges matched the calculated advancement, and there was no significant difference between the measured value and the wedge size (*p* = 0.499). The mean difference between the calculated advancement and the wedge selected was 0.089 mm for TAM.

The postoperative PTA was 90.1° ± 3.7° for the advancements measured by TAM and 88.8° ± 4.8° for CT, and it was not significantly different between the two methods (*p* = 0.622). For eight stifle joints assessed using TAM, the postoperatively measured PTA was lower than the preoperatively measured value; the value increased after surgery for two stifles. Using CT, the PTA decreased after MMP in nine stifles and increased in one. There were no stifles in which the postoperative PTA was identical to the value measured preoperatively for surgery for either method.

There was no significant difference between the preoperative and postoperative PTAs after using TAM (*p* = 0.059). However, there was a significant difference between the preoperative and postoperative PTAs after using CT (*p* = 0.007).

The positions of the wedges relative to the Maquet hole were correct for both methods according to the surgical procedure proposed by Ness [22] with a mean Mq-W value of 4.7 ± 1.9 mm. The mean distance between the proximal edge of the wedge and the insertion point of the patellar tendon was 12.1 ± 2.6 mm. The mean Mq-W determined after TAM and CT were 3.9 ± 2.0 and 5.3 ± 1.6 mm, respectively. The distribution of Mq-W was similar for both methods (*p* = 0.298). The mean W-PT was 11.2 ± 2.9 mm for TAM and 12.9 ± 2.2 mm for CT, and it was not significantly different between the two methods (*p* = 0.245).

## 4. Discussion

This prospective cadaveric study evaluated the effectiveness of using TAM or CT for the preoperative planning of MMP to achieve a postoperative target PTA of 90°.

Numerous adaptations of TTA have been described and include the modified Maquet technique [20], rapid TTA [24], MMP [22], modified Maquet tibial tuberosity advancement [36], tibial tuberosity advancement with cranial fixation [37], and porous TTA [38]. The tibial tuberosity advancement and its further adaptations, such as MMP, are widely used to neutralize the cranial tibial thrust in dogs with cranial cruciate ligament failure. The satisfactory clinical outcomes of the MMP method prompted us to investigate the preoperative planning for this procedure [13,16,22]. A preoperative assessment of TTA is a mandatory step for MMP and for TTAT in general. The goal of preoperative planning is to predict TTA accurately and achieve a final PTA of 90° by selecting the correctly sized wedge.

In our study, the advancement determined by TAM and CT did not differ significantly between these two methods. This result was consistent with a previous study that found no difference between advancement values determined with TAM and CT [34]. However, PTA was not reported, preventing an objective comparison.

Using CT, we found a statistically significant difference (*p* = 0.013) between the preoperatively measured advancement and the size of the wedges chosen intraoperatively. Using CT, the tibial tuberosity advancement required was <5.3 mm for 60% of the stifles, but the smallest commercially available wedge size is 6 mm. Consequently, 6 mm wedges were used in the stifles in which the calculated advancement was <6 mm. The mean PTA after surgery was 88° for CT, but this value should be critically interpreted considering the restriction of the available wedge sizes.

By contrast, using TAM, the measured size of the wedge and the size of the commercially available wedge were overlapped with a very small mean difference. This measurement technique can improve the accuracy of wedge selection in clinical practice. Indeed, the final PTA after TAM was close to the target of 90°. This demonstrates the low discrepancy between the preoperatively measured advancement and the intraoperatively achieved value when using TAM.

The final PTA was within the target range for a satisfactory clinical outcome for 80% of the cases. This result was consistent with the data reported by Della Valle et al., who reported a mean final PTA of 89.7° following MMP in a sample of 35 dogs [16]. Conversely, in 2015, Kapler and colleagues reported that when using TAM and the modified tibial tuberosity advancement method, only 53% of the procedures resulted in a PTA within 90° ± 5 [21].

The lack of a significant difference in the final PTA between the two methods was due to the selection of overlapping wedge sizes. The commercially available wedge sizes, which differed in size by 1.5 mm, could have influenced these findings because only a significant difference between the two methods would have caused a marked change in the selected wedge size.

This prospective study investigated CT because there have been no prior reports describing the use of this method for selecting the appropriate wedge size to achieve the desired advancement. Although the interobserver reliability was poor in previous studies, CT is the most commonly used method in the preoperative planning of TTAT, followed by TAM [13]. The common tangent method is based on the evidence that the tibial thrust is neutral when the patellar tendon is perpendicular to the tibial plateau [14] and on the assumption that this should be achieved at a stifle angle of 135°, which resembles the mid-stance phase of the gait cycle [35]. This method disregards the need for the TPA to determine the necessary advancement.

We used TAM based on the results of a study of the currently available literature [34]. The TAM method relies on tibial landmarks exclusively [22] and does not require the stifle to be positioned at an angle of 135° for radiography, avoiding the inaccuracy created by tibial subluxation, as demonstrated by Bielecki et al. [19]. On the other hand, it appeared to underestimate the size of the wedge needed to provide the desired advancement, as reported by Kapler and colleagues [21].

Radiographic methods described for the determination of the TTA include the conventional method [39], a correction method [20], CT [35], TAM [22], the modified tibial tuberosity advancement method [21], the Bielecki method [19], and the osteotomy axis method [23]. Most of the available measurement methods were developed for traditional TTA [14,20,35,39], which is characterized by a different direction of advancement than TTAT. Moreover, several papers have demonstrated the ineffectiveness of these methods for obtaining adequate advancement [15,25,39]. Therefore, we decided not to investigate those techniques further.

Although the previously described methods were associated with good clinical outcomes in some studies [20,22], other studies suggested that these methods did not accurately determine the advancement required to achieve the target PTA of 90° [21,23]. Historically, a PTA of 90° ± 5° has been considered sufficient to neutralize the tibiofemoral shear force [25], but a suboptimal postoperative PTA and the resulting instability may explain the frequency of late meniscal tears after a TTAT of 4.3% [13]. This percentage is lower than that of 28% reported for traditional TTA [40].

Furthermore, in 2013, Skinner et al. reported that 70% of dogs with a mean PTA assessed using a CT (PTA_CT_) of 89° after traditional TTA showed persistent cranial tibial subluxation [29]. This assumed critical point may vary among breeds or might be subject to individual factors. However, it is unlikely to affect the functional outcome after TTAT that is acceptable in most dogs [13]. The method used to determine PTA seems to influence the measurement of the required advancement, but there are no definitive recommendations in the veterinary literature regarding which method should be used to assess PTA. Moreover, poor agreement between PTA measured with the tibial plateau method and PTA_CT_ has been widely reported [28,41,42,43]. We determined PTA_TP_ on preoperative radiographs because it is commonly used in our clinic and because it demonstrated better intra- and interobserver reliability than PTA_CT_ [28]. However, by enrolling healthy dogs with similar morphological characteristics, the preoperative PTAs were comparable with both methods, minimising variability.

Another source of error is related to the discrepancy between the line passing through the distal PT insertion, where advancement is measured, and the line corresponding to the base of the wedge. Because the wedge is trapezoidal, its size corresponds to its base. Therefore, if the proximal edge of the wedge is not at the level of the PT insertion, the true advancement could differ from the preoperatively determined dimension. The comparable positions of the wedges using both methods showed that we minimized any bias caused by this variable. Despite this similarity, the position of the wedge inside the osteotomy may have influenced the postoperative PTA for both methods. However, as previously explained, this difference was probably compensated for by rounding up the calculated advancement to the wedge size [44].

To our knowledge, there are no published reports describing the position of the wedge for MMP. By comparison, the distance between the proximal edge of the osteotomy and the cage should be between 3 and 5 mm, as currently recommended for rapid TTA [24]. However, the cage used in this surgical technique is significantly different from that designated for MMP, which means this advice is unsuitable. However, in the present study, the average Mq-W was within this range.

Moreover, in the present study, the mean distance between the distal insertion of the PT and the proximal edge of the wedge was 12 mm for both methods. Therefore, if the line passing through the proximal edge of the wedge does not match the line of the PT, the measured advancement does not correspond to the wedge size. This may explain why previous studies have not consistently determined the true advancement by MMP [21]. This was recently confirmed for traditional TTA, where recommendations for implant design and cage position resulted in under-advancements of 15% [27], 21–28% [26], and 30% [21].

This study has some limitations, one being the number of stifles included (n = 20) due to the inclusion criteria. However, we sought to reduce the variability between the two methods by randomly assigning the limbs of the same dog to each method. This allowed us to test the two preoperative measurement techniques in limbs with overlapping anatomical features.

## 5. Conclusions

No definitive recommendation has been published in the currently available literature for a preoperative planning method to achieve a PTA corresponding to 90°. Even though the postoperative PTA in our sample was not significantly different between the two methods, TAM, according to the results of this study, achieved the desired PTA of 90° in the majority of the presented cases.

In our experience, TAM is easier to perform as it avoids errors due to the mispositioning of the legs and provides advancement values of the tibial tuberosity that are generally consistent with the sizes of commercially available wedges. By comparison, even though CT yielded tibial tuberosity advancement measurements that were not statistically different from those provided by TAM, CT failed to achieve the target of 90°, and thus it is not recommended as part of the preoperative planning of MMP. Considering the preoperative and intraoperative variables that may affect the true advancement, the development of an intraoperative method that can reliably measure the desired tibial tuberosity advancement of 90° is necessary to improve the clinical effectiveness of the TTAT.

## Data Availability

All datasets generated for this study are included in the article.

## References

[1] Witsberger T.H., Villamil J.A., Schultz L.G., Hahn A.W., Cook J.L. (2008). Prevalence of and risk factors for hip dysplasia and cranial cruciate ligament deficiency in dogs. J. Am. Vet. Med. Assoc..

[2] Ichinohe T., Kanno N., Harada Y., Yogo T., Tagawa M., Hara Y. (2015). Histological and immunohistological analysis of degenerative changes in the cranial cruciate ligament in a canine model of excessive tibial plateau angle. Vet. Comp. Orthop. Traumatol..

[3] Baker L.A., Kirkpatrick B., Rosa G.J.M., Gianola D., Valente B., Sumner J.P., Baltzer W., Hao Z., Binversie E.E., Volstad N. (2017). Genome-wide association analysis in dogs implicates 99 loci as risk variants for anterior cruciate ligament rupture. PLoS ONE.

[4] Wilke V.L., Zhang S., Evans R.B., Conzemius M.G., Rothschild M.F. (2009). Identification of chromosomal regions associated with cranial cruciate ligament rupture in a population of Newfoundlands. Am. J. Vet. Res..

[5] Ragetly C.A., Evans R., Mostafa A.A., Griffon D.J. (2011). Multivariate analysis of morphometric characteristics to evaluate risk factors for cranial cruciate ligament deficiency in Labrador retrievers. Vet. Surg..

[6] Duval J.M., Budsberg S.C., Flo G.L., Sammarco J.L. (1999). Breed, sex, and body weight as risk factors for rupture of the cranial cruciate ligament in young dogs. J. Am. Vet. Med. Assoc..

[7] Restucci B., Sgadari M., Fatone G., Valle G.D., Aragosa F., Caterino C., Ferrara G., Niebauer G.W. (2022). Immunoexpression of Relaxin and Its Receptors in Stifle Joints of Dogs with Cranial Cruciate Ligament Disease. Animals.

[8] Bleedorn J.A., Greuel E.N., Manley P.A., Schaefer S.L., Markel M.D., Holzman G., Muir P. (2011). Synovitis in dogs with stable stifle joints and incipient cranial cruciate ligament rupture: A cross-sectional study. Vet. Surg..

[9] Little J.P., Bleedorn J.A., Sutherland B.J., Sullivan R., Kalscheur V.L., Ramaker M.A., Schaefer S.L., Hao Z., Muir P. (2014). Arthroscopic assessment of stifle synovitis in dogs with cranial cruciate ligament rupture. PLoS ONE.

[10] Fuller M.C., Hayashi K., Bruecker K.A., Holsworth I.G., Sutton J.S., Kass P.H., Kantrowitz B.J., Kapatkin A.S. (2014). Evaluation of the radiographic infrapatellar fat pad sign of the contralateral stifle joint as a risk factor for subsequent contralateral cranial cruciate ligament rupture in dogs with unilateral rupture: 96 cases (2006–2007). J. Am. Vet. Med. Assoc..

[11] Taylor-Brown F.E., Meeson R.L., Brodbelt D.C., Church D.B., McGreevy P.D., Thomson P.C., O’Neill D.G. (2015). Epidemiology of cranial cruciate ligament disease diagnosis in dogs attending primary-care veterinary practices in England. Vet. Surg..

[12] Vaughan L.C. (2010). The history of canine cruciate ligament surgery from 1952–2005. Vet. Comp. Orthop. Traumatol..

[13] Aragosa F., Caterino C., Della Valle G., Fatone G. (2022). Tibial Tuberosity Advancement Techniques (TTAT): A Systematic Review. Animals.

[14] Montavon P.M. Advancement of the tibial tuberosity for the treatment of cranial cruciate deficient canine stifle. Proceedings of the 1st World Orthopaedic Veterinary Congress.

[15] Pillard P., Livet V., Cabon Q., Bismuth C., Sonet J., Remy D., Fau D., Carozzo C., Viguier E., Cachon T. (2016). Comparison of desired radiographic advancement distance and true advancement distance required for patellar tendon–tibial plateau angle reduction to the ideal 90° in dogs by use of the modified Maquet technique. Am. J. Vet. Res..

[16] Della Valle G., Caterino C., Aragosa F., Micieli F., Costanza D., Di Palma C., Piscitelli A., Fatone G. (2021). Outcome after Modified Maquet Procedure in dogs with unilateral cranial cruciate ligament rupture: Evaluation of recovery limb function by use of force plate gait analysis. PLoS ONE.

[17] Backstein D., Meisami B., Gross A.E. (2003). Patella baja after the modified Coventry-Maquet high tibial osteotomy. J. Knee Surg..

[18] Neville-Towle J.D., Makara M., Johnson K.A., Voss K. (2016). Effect of proximal translation of the osteotomized tibial tuberosity during tibial tuberosity advancement on patellar position and patellar ligament angle. BMC Vet. Res..

[19] Bielecki M.J., Schwandt C.S., Scharvogel S. (2014). Effect of tibial subluxation on the measurements for tibial tuberosity advancement in dogs with cranial cruciate ligament deficiency: An ex vivo study. Vet. Comp. Orthop. Traumatol..

[20] Etchepareborde S., Brunel L., Bollen G., Balligand M. (2011). Preliminary experience of a modified maquet technique for repair of cranial cruciate ligament rupture in dogs. Vet. Comp. Orthop. Traumatol..

[21] Kapler M.W., Marcellin-Little D.J., Roe S.C. (2015). Planned wedge size compared to achieved advancement in dogs undergoing the modified Maquet procedure. Vet. Comp. Orthop. Traumatol..

[22] Ness M.G. (2016). The Modified Maquet Procedure (MMP) in Dogs: Technical Development and Initial Clinical Experience. J. Am. Anim. Hosp. Assoc..

[23] Pillard P., Livet V., Cabon Q., Bismuth C., Sonet J., Remy D., Fau D., Carozzo C., Viguier E., Cachon T. (2017). Evaluation of a new method to determine the tibial tuberosity advancement distance required to reduce the patellar tendon-tibial plateau angle to 90° with the modified Maquet technique in dogs. Am. J. Vet. Res..

[24] Samoy Y., Verhoeven G., Bosmans T., Van der Vekens E., de Bakker E., Verleyen P., Van Ryssen B. (2015). TTA rapid: Description of the technique and short term clinical trial results of the first 50 cases. Vet. Surg..

[25] Etchepareborde S., Mills J., Busoni V., Brunel L., Balligand M. (2011). Theoretical discrepancy between cage size and efficient tibial tuberosity advancement in dogs treated for cranial cruciate ligament rupture. Vet. Comp. Orthop. Traumatol..

[26] Jin D.W., Peck J.N., Tano C.A., Morgan M.J. (2019). Discrepancy between true distance of tibial tuberosity advancement and cage size: An ex vivo study. Vet. Surg..

[27] Meeson R.L., Corah L., Conroy M.C., Calvo I. (2018). Relationship between Tibial conformation, cage size and advancement achieved in TTA procedure. BMC Vet. Res..

[28] Millet M., Bismuth C., Labrunie A., Marin B., Filleur A., Pillard P., Sonet J., Cachon T., Etchepareborde S. (2013). Measurement of the patellar tendon-tibial plateau angle and tuberosity advancement in dogs with cranial cruciate ligament rupture. Vet. Comp. Orthop. Traumatol..

[29] Skinner O.T., Kim S.E., Lewis D.D., Pozzi A. (2013). In vivo femorotibial subluxation during weight-bearing and clinical outcome following tibial tuberosity advancement for cranial cruciate ligament insufficiency in dogs. Vet. J..

[30] Bush M.A., Bowlt K., Gines J.A., Owen M.R. (2011). Effect of use of different landmark methods on determining stifle angle and on calculated tibial tuberosity advancement. Vet. Comp. Orthop. Traumatol..

[31] Giansetto T., Picavet P.P., Lefebvre M., Balligand M. (2022). Determination of the Stifle Angle at Standing Position in Dogs. Vet. Sci..

[32] Ševčík K., Hluchý M., Ševčíková M., Ledecký V. (2021). Radiographic measurement of canine stifle joint angles using four different landmark methods. Acta Vet. Brno.

[33] Apelt D., Kowaleski M.P., Boudrieau R.J. (2007). Effect of tibial tuberosity advancement on cranial tibial subluxation in canine cranial cruciate-deficient stifle joints: An in vitro experimental study. Vet. Surg..

[34] Della Valle G., Caterino C., Piscitelli A., Di Palma C., Pasolini M.P., Lamagna F., Muto A., Fatone G. Reliability of three different methods to measure the amount of tibial tuberosity advancement in the preoperative planning of modified maquet precedure. Proceedings of the 73rd Scientific Meeting of the Italian Veterinary Sciences Society (SISVet 2019).

[35] Dennler R., Kipfer N.M., Tepic S., Hassig M., Montavon P.M. (2006). Inclination of the patellar ligament in relation to flexion angle in stifle joints of dogs without degenerative joint disease. Am. J. Vet. Res..

[36] Medeiros R.M., Silva M.A.M., Teixeira P.P.M., Dias L., Chung D.G., Zani C.C., Feliciano M.A.R., Da Conceicao M., Machado M.R.F., Rocha A.G. (2016). Use of castor bean polymer in developing a new technique for tibial tuberosity advancement for cranial cruciate ligament rupture correction in dogs. Vet. Med..

[37] Zhalniarovich Y., Sobolewski A., Waluś G., Adamiak Z. (2018). Evaluation, Description of the Technique, and Clinical Outcomes After Tibial Tuberosity Advancement with Cranial Fixation (TTA CF) for Cranial Cruciate Ligament Rupture in 22 Dogs. Top. Companion Anim. Med..

[38] Trisciuzzi R., Fracassi L., Martin H.A., Monopoli Forleo D., Amat D., Santos-Ruiz L., De Palma E., Crovace A.M. (2019). 41 Cases of Treatment of Cranial Cruciate Ligament Rupture with Porous TTA: Three Years of Follow Up. Vet. Sci..

[39] Cadmus J., Palmer R.H., Duncan C. (2014). The Effect of Preoperative Planning Method on Recommended Tibial Tuberosity Advancement Cage Size. Vet. Surg..

[40] Wolf R.E., Scavelli T.D., Hoelzler M.G., Fulcher R.P., Bastian R.P. (2012). Surgical and postoperative complications associated with tibial tuberosity advancement for cranial cruciate ligament rupture in dogs: 458 cases (2007–2009). J. Am. Vet. Med. Assoc..

[41] Boudrieau R.J. (2009). Tibial plateau leveling osteotomy or tibial tuberosity advancement?. Vet. Surg..

[42] Hoffmann D.E., Kowaleski M.P., Johnson K.A., Evans R.B., Boudrieau R.J. (2011). Ex vivo biomechanical evaluation of the canine cranial cruciate ligament-deficient stifle with varying angles of stifle joint flexion and axial loads after tibial tuberosity advancement. Vet. Surg..

[43] Schwandt C.S., Bohorquez-Vanelli A., Tepic S., Hassig M., Dennler R., Vezzoni A., Montavon P.M. (2006). Angle between the patellar ligament and tibial plateau in dogs with partial rupture of the cranial cruciate ligament. Am. J. Vet. Res..

[44] Butterworth S.J., Kydd D.M. (2017). TTA-Rapid in the treatment of the canine cruciate deficient stifle: Short-and medium-term outcome. J. Small Anim. Pract..

